# Mechanobiological Approach to Design and Optimize Bone Tissue Scaffolds 3D Printed with Fused Deposition Modeling: A Feasibility Study

**DOI:** 10.3390/ma13030648

**Published:** 2020-02-01

**Authors:** Gianluca Percoco, Antonio Emmanuele Uva, Michele Fiorentino, Michele Gattullo, Vito Modesto Manghisi, Antonio Boccaccio

**Affiliations:** Dipartimento di Meccanica, Matematica e Management, Politecnico di Bari, Via E. Orabona 4, 70126 Bari, Italy; gianluca.percoco@poliba.it (G.P.); antonio.uva@poliba.it (A.E.U.); michele.fiorentino@poliba.it (M.F.); michele.gattullo@poliba.it (M.G.); vitomodesto.manghisi@poliba.it (V.M.M.)

**Keywords:** tissue engineering, biomaterials, mechanobiology, scaffold design, geometry optimization

## Abstract

In spite of the rather large use of the fused deposition modeling (FDM) technique for the fabrication of scaffolds, no studies are reported in the literature that optimize the geometry of such scaffold types based on mechanobiological criteria. We implemented a mechanobiology-based optimization algorithm to determine the optimal distance between the strands in cylindrical scaffolds subjected to compression. The optimized scaffolds were then 3D printed with the FDM technique and successively measured. We found that the difference between the optimized distances and the average measured ones never exceeded 8.27% of the optimized distance. However, we found that large fabrication errors are made on the filament diameter when the filament diameter to be realized differs significantly with respect to the diameter of the nozzle utilized for the extrusion. This feasibility study demonstrated that the FDM technique is suitable to build accurate scaffold samples only in the cases where the strand diameter is close to the nozzle diameter. Conversely, when a large difference exists, large fabrication errors can be committed on the diameter of the filaments. In general, the scaffolds realized with the FDM technique were predicted to stimulate the formation of amounts of bone smaller than those that can be obtained with other regular beam-based scaffolds.

## 1. Introduction

The development of the recent additive manufacturing techniques and, consequently, the possibility of building constructs with very sophisticated geometries, led many researchers to investigate the scaffold geometries that mostly favor the formation of bone in the shortest time. To this purpose, both regular and irregular scaffold geometries were proposed and investigated. The regular scaffolds include unit cell configurations all with the same shape and dimensions that are regularly replicated in the scaffold volume [1,2]. Analytical solutions were recently developed that put in relationship the equivalent material properties of the entire scaffold with the dimensions and the material properties of the single unit cell [3,4,5,6]. The irregular ones include pores differently shaped and dimensioned and present a geometry that can be described with statistical parameters [7,8,9] but not in a precise form [10]. Although irregular geometry scaffolds are commonly utilized in experiments for bone tissue engineering, regular scaffolds have gained importance in recent decades as they allow precise control of the actual geometry and are, hence, suited to create repeatable physical environments and easier to investigate [5]. Among the possible geometries of regular scaffolds, the geometry realized by means of the fused deposition modeling technique certainly represents an important solution.

The fabrication of scaffolds with additive manufacturing techniques is an issue that recently received recognition and attention from the research community [11,12]. Different rapid prototyping techniques were proposed to fabricate biomaterials scaffolds, based on liquid polymerization [13], material deposition processes [14], powder-based processes [15], sheet lamination [16], binder jetting [17], and material jetting [18]. Among the other rapid prototyping techniques, fused deposition modeling (FDM) is one of the most common 3D printing technologies available on the market, thanks to its low cost, ease of use, and the variety of usable materials. It consists in depositing layers of a polymeric, ceramic, or metallic material; each layer includes cylindrical strands all oriented in a given direction and equally spaced. To guarantee an adequate structural response, the orientation of filaments changes layer by layer. Furthermore, between the cylindrical filaments of adjacent layers, an overlap region exists where the single strand is melted with the adjacent one during the deposition process. Recently, this technique has been successfully utilized to fabricate scaffolds for bone tissue engineering [19,20,21,22,23]. Process parameters of the FDM technique were also optimized to improve the dimensional accuracy of the manufactured components [24]. The typical/traditional approach consists in using the FDM technique by implementing standard process parameters that allow obtaining accurate structures. However, adjusting the strand diameter, and modifying the process parameters is a challenging task and still remains a research topic. In fact, when small modifications on the scaffold geometry must be achieved, acting on process parameters of the traditional FDM technique can be a valid option instead of looking for expensive, more accurate technologies. A recent study proposed a numerical model to simulate the extrusion of a strand of semi-molten material to investigate how the strand cross-section changes for variable process parameters [25]. The porosity and the micro-architecture of parts built with the FDM technique appear very suited to stimulate the colonization and subsequent differentiation of mesenchymal stem cells [26]. 

Optimization algorithms were implemented to improve the scaffold performance and to minimize the negative effects related to the implantation of the scaffold in the fracture site [27]. Many objective functions were investigated and different optimization strategies were implemented [28,29,30,31]. Most of the optimization studies reported in the literature aim to minimize the difference between the equivalent mechanical properties of the scaffold and those of the tissue within which it is implanted. In one word, such studies that consider scaffold stiffness as the design variable aim to minimize the effects of stress shielding at the bone/scaffold interface [32,33]. Optimization strategies based on the compressive modulus expressed as a ratio between third principal stress and the prescribed compressive strain were also utilized to optimize the geometry of scaffolds fabricated with the FDM technique [34]. Singh et al. have proposed a multifactor optimization for the development of biocompatible and biodegradable composite material-based feedstock filament of fused deposition modeling [35]. Only recently, optimization algorithms based on mechanobiological criteria were proposed to design and optimize small volumes of scaffolds with both, regular [36,37,38,39,40] and irregular [41] micro-geometry [42]. In these mechanobiology-based optimization algorithms, the scaffold geometry is perturbed until the micro-architecture that allows maximizing the formation of new bone, is identified. However, to the knowledge of the present authors, no studies are reported in the literature that optimize the geometry of scaffolds fabricated with the FDM technique and that are based on mechanobiological criteria. In this study, we want to bridge this gap. 

The objective of this feasibility study is to optimize the geometry of scaffolds fabricated with the FDM technique and to investigate whether this technique can be utilized to fabricate scaffolds designed and optimized to undergo a compression load. The objective function that was utilized is based on the computational mechano-regulation algorithm by Prendergast and Huiskes [43,44]. Such an algorithm models the fracture domain as a biphasic poroelastic material, and hypothesizes the biophysical stimulus that triggers the tissue differentiation process to be a function of the octahedral shear strain and of the fluid velocity. This mechanobiological algorithm was successfully utilized in previous studies investigating the healing process in fractured bones [45,46], in osteochondral defects [47], at the implant/bone interfaces [48] and the regeneration process in scaffolds for bone tissue engineering [49,50]. 

Other mechano-regulation computational models are reported in the literature, investigating the role of the mechanical environment on the biophysical stimulus that triggers the tissue differentiation process [51,52,53,54]. However, the patterns of tissue differentiation predicted by the model of Prendergast and Huiskes were shown to be closest to experimental results compared to other mechanobiological algorithms [55].

We determined, for different values of the filament diameter, the optimal distance between the strands that, for the specific load acting on the scaffold, can maximize the formation of bony tissue. The optimized scaffolds were physically fabricated and successively measured. By utilizing a CMM machine, the distance between the filaments and the filament diameter were measured with high accuracy and compared with the corresponding nominal values. 

## 2. Materials and Methods 

### 2.1. Parametric Finite Element Model

**Figure 1:**
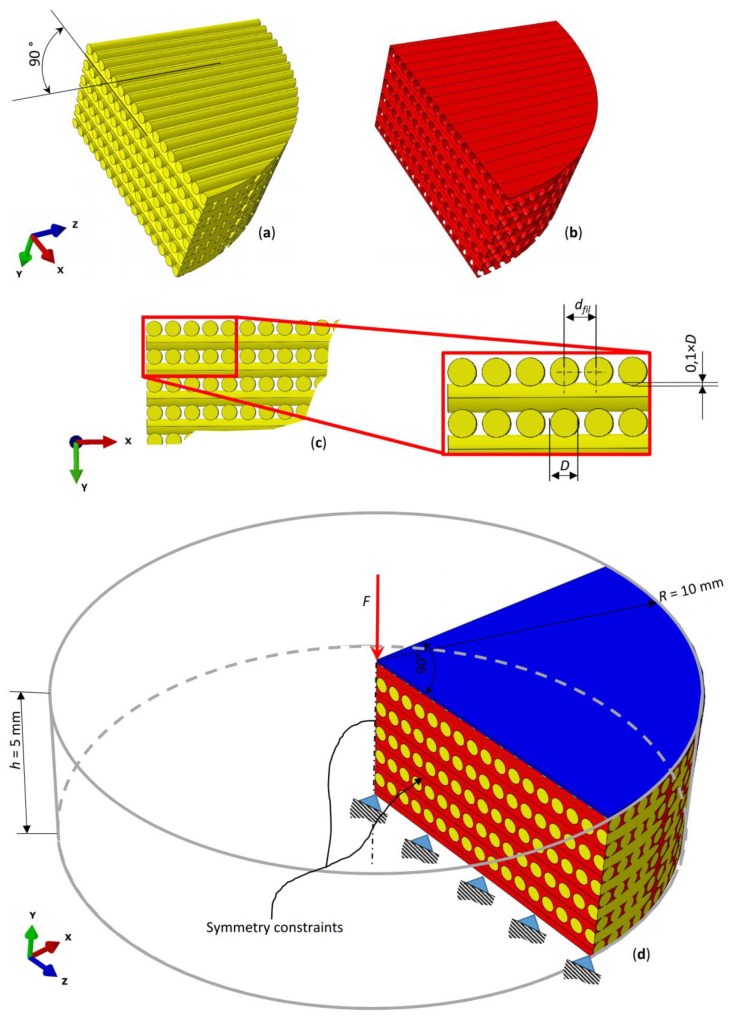
Parametric model of the scaffold (**a**,**c**) and of the granulation tissue (**b**) occupying the scaffold pores. (**d**) Exploiting the symmetry of the system, the one-quarter model was investigated.

**Figure 2:**
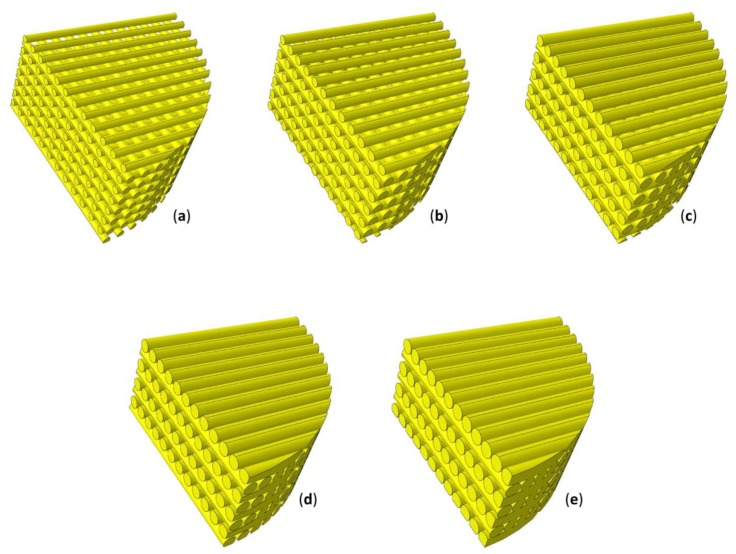
Models of scaffold investigated in the study including strands with diameter *D* = 400 µm (**a**), 500 µm (**b**), 600 µm (**c**), 700 µm (**d**), and 800 µm (**e**). The distance between the filaments was computed by means of the proposed optimization algorithm.

The parametric poroelastic finite element model of a cylindrical scaffold with radius *R* = 20 mm and *h* = 5 mm high was built in Abaqus^®^ (version 6.12, Dassault Systèmes, Vélizy-Villacoublay, France) (Figure 1). Scaffolds with the same dimensions were utilized by Teng et al. [56]. The model consists of layers including aligned cylindrical strands with diameter *D* equally spaced. The filaments of two adjacent layers form an angle of 90° (Figure 1a). Five different values were considered for the diameter *D*: 400, 500, 600, 700, and 800 µm while the distance between the filaments *d_fil_* was optimized via the optimization algorithm described below (Figure 2). Following Somireddy and Czekanski [57], between two adjacent layers, an overlap region of 0.1 × *D* was hypothesized. The model of the tissue occupying the scaffold pores (highlighted in red in Figure 1) was built by means of Boolean operations of subtraction, from the entire model volume *V_TOT_* = π × *R*^2^ × *h*, the volume of the scaffold. 

**Figure 3:**
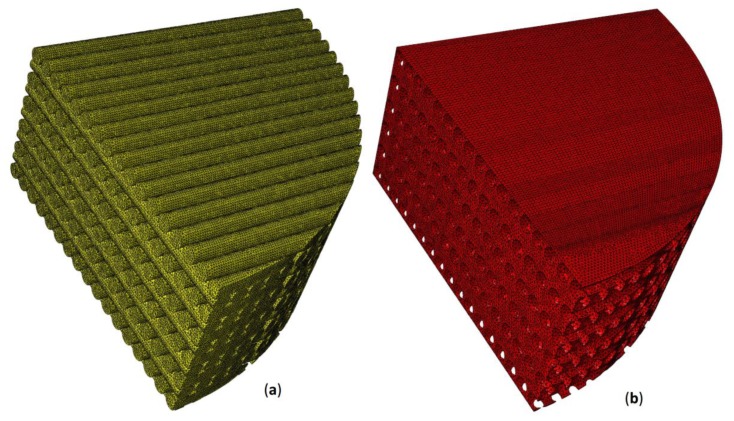
Finite element mesh utilized to model the scaffold (**a**) and the granulation tissue (**b**) including poroelastic four-node tetrahedral elements (C3D4P).

According to previous studies [38,58], the volume inside the pores was hypothesized to be occupied by granulation tissue. Exploiting the symmetry of the system, to reduce the computational cost, a one-quarter model was developed. The lower base of the scaffold was clamped while a compression load was applied on the upper surface by means of a rigid plate (highlighted in blue in Figure 1). Different values of the compression load *F* (Figure 1) were applied to the model, corresponding to the following values of force per unit surface *p* = 0.2, 0.5, 1.0, and 1.5 MPa. Such values are consistent with those hypothesized in previous studies [36,58]. Symmetry constraints were applied on the lateral surfaces to simulate the continuity of the entire model (Figure 1). Poroelastic four-node tetrahedral finite elements C3D4P available in Abaqus were adopted to mesh the scaffold model (Figure 3). The model of the scaffold and of the granulation tissue included about 5M elements with 1M nodes. 

The modeled scaffolds were physically fabricated via the FDM technique by utilizing the polylactic acid (PLA), a biodegradable thermoplastic polyester considered a bioplastic, possessing a Young’s modulus of 2300 MPa [59]. This same value of Young’s modulus was implemented in the finite element model of the scaffold and utilized in the optimization algorithm described below. The other material properties implemented for scaffold and granulation tissue are the same as those utilized in previous studies [36,40,60] and are listed in Table 1.

### 2.2. Mechanobiological Model by Prendergast and Huiskes to Describe the Bone Regeneration Process inside the Scaffold 

Once the scaffold is implanted in the anatomic region with bone deficiency, the mesenchymal stem cells (MSCs) migrate from the surrounding tissues and invade the scaffold pores. Based on the mechanical stimulus acting on them, MSCs will start to differentiate into different phenotypes. The mechanobiological model by Prendergast and Huiskes hypothesizes that the biophysical stimulus *S* that triggers the tissue differentiation process is a function of the octahedral shear strain *γ* and of the interstitial fluid flow *v*, i.e., the velocity with which the fluid flows through the solid phase, according to the relationship

(1) $$S=\frac{\gamma }{a}+\frac{v}{b}$$

where *a* = 3.75 % and *b* = 3 μm/s are two empirical constants determined in a previous study [43]. In particular, the octahedral shear strain *γ* can be expressed in function of the principal strains *ε_I_*, *ε_II_* and *ε_III_* as

(2) $$\gamma =\frac{2}{3}\sqrt{{\left(\varepsilon_{I}-\varepsilon_{II}\right)}^{2}+{\left(\varepsilon_{II}-\varepsilon_{III}\right)}^{2}+{\left(\varepsilon_{I}-\varepsilon_{III}\right)}^{2}}$$

Depending on the value of *S*, stem cells will be differentiated into the following phenotypes

(3) $$\{\begin{array}{c} if\;S\;>\;3\;\to \;formation\;of\;fibroblasts\;\left(Fibrous\;tissue\right)\\ else\;if\;1\;<\;S\;<\;3\;\to \;formation\;of\;chondrocytes\;\left(Cartilage\;tissue\right)\\ else\;if\;0.53\;<\;S\;<\;1\;\to formation\;of\;osteoblasts\;\left(Immature\;bone\right)\\ else\;if\;0.01\;<\;S\;<\;0.53\;\to \;formation\;of\;osteoblasts\;\left(Mature\;bone\right)\\ else\;if\;0\;<\;S\;<\;0.01\;\to \;bone\;resorption \end{array}$$

The threshold limits reported in the inequalities (3) are the same as those utilized in a previous study [61].

### 2.3. Mechanobiology-Based Optimization Algorithm

**Figure 4:**
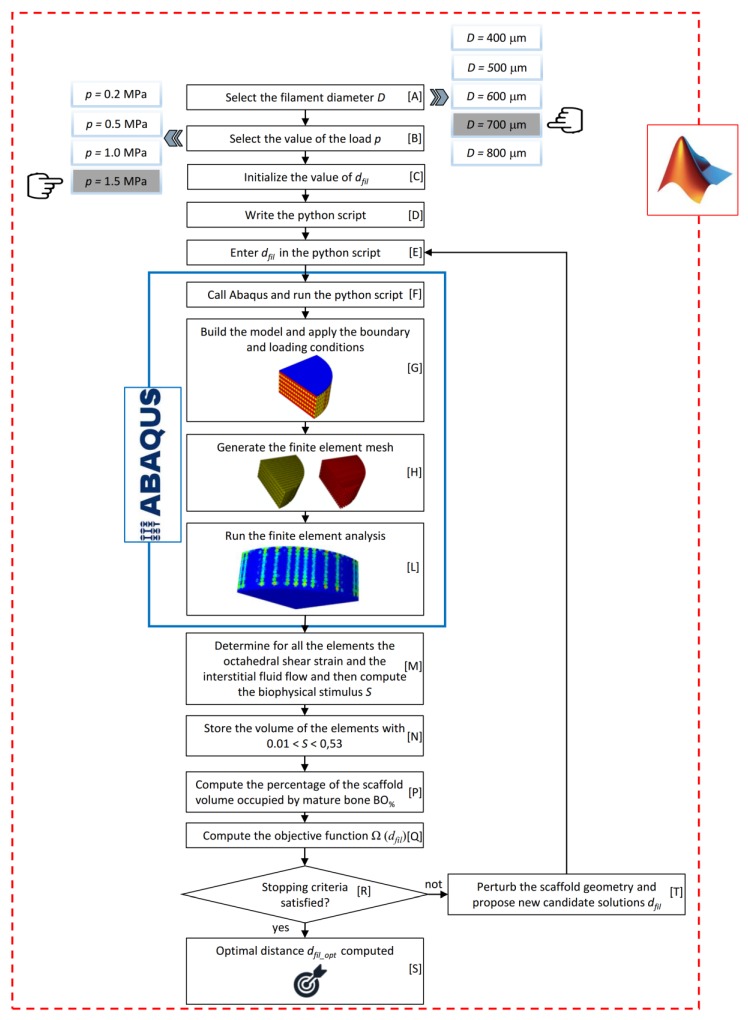
Schematic of the optimization algorithm written in MATLAB environment.

The task of determining the optimal distance *d_fil_* between the filaments was accomplished by implementing an optimization algorithm, a schematic of which is illustrated in Figure 4. The choice of utilizing *d_fil_* as a design variable and the strand diameter *D* as input parameter entered by the user derives from the fact that in the FDM technique, the distance between the strands can be changed with continuity while the strand diameter cannot be controlled with precision as it depends on the nozzle diameter. The algorithm implements the optimization tool available in MATLAB^®^ (Version R2016b, MathWorks, Natick, USA) *fmincon* devoted to finding the minimum of a multivariable scalar function starting at an initial estimate. The objective of the optimization algorithm is to identify the optimal value of the filaments distance *d_fil_optim_* that allows maximizing the amounts of mature bone that are predicted to generate within the scaffold pores.

The user has, first, to select the value of the diameter *D* and of the load per unit area *p* acting on the scaffold model (Blocks [A] and [B], Figure 4). Then, the user is prompted to specify a tentative initial value of the distance between the filaments *d_fil_* (Block [C], Figure 4). At his point, the algorithm writes a python script (Block [D]) and enters into it the tentative value provided by the user (Block [E], Figure 4). The python script is then given in input to Abaqus (Block [F], Figure 4) that, executing the instructions of the script: (i) builds the CAD model of both, the scaffold and the granulation tissue and applies the boundary and loading conditions above described (Figure 1d) (Block [G], Figure 4); (ii) generates the poroelastic tetrahedral finite element mesh (Block [H], Figure 4); and (iii) runs the finite element (FE) analysis (Block [L], Figure 4). Once the FE analysis has terminated, Abaqus prints, for all the elements inside the scaffold pores (highlighted in red in Figure 1), the volume of the element and the values of strain and interstitial fluid velocity computed in the analysis. At this point, the algorithm reads the document printed by Abaqus and computes, according to the Equation (1) the value of the biophysical stimulus *S* acting on the single element (Block [M], Figure 4). Then, the algorithm compares all the obtained values of *S* with the threshold limit reported in the inequalities (3). For the elements where the formation of mature bone is predicted (i.e., the inequality 0.01 < *S* < 0.53 is satisfied) the algorithm stores the value of the element volume (Block [N], Figure 4). 

**Figure 5:**
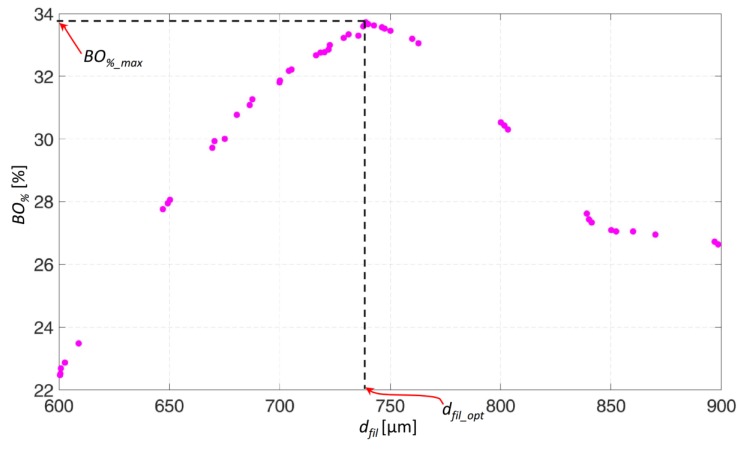
Typical values of *BO_%_* obtained in an optimization process for different values of *d_fil_*. In detail, the diagram refers to the case of *D* = 600 µm and *p* = 0.5 MPa.

Once for all the elements, *S* was computed, the algorithm determines the total volume of the elements that were predicted to differentiate into mature bone, by summing up all the element volumes previously stored. Then, if *V_BONE_* is the total volume of the elements ‘mature bone’ and *V_TOT_* = π × *R*^2^ × *h* the total volume of the model, the algorithm computes the percentage *BO_%_* of the scaffold volume that is predicted to be occupied by mature bone as the ratio between *V_BONE_* and *V_TOT_* multiplied by 100 (Block [P], Figure 4). As it is clear, the task pursued by the algorithm is to increase as much as possible the percentage *BO_%_*. However, considering that the optimization tool utilized *fmincon* is designed to determine the minimum value of functions, the objective function Ω(*d_fil_*) was defined as the opposite value of *BO_%_* (Block [Q], Figure 4). At this point, the algorithm perturbs the scaffold geometry many times (Block [R], Figure 4), i.e., it proposes different values of *d_fil_* as possible candidate solutions (Block [T], Figure 4) and for each of the proposed value, it stores the value of Ω(*d_fil_*). Figure 5 shows, for instance, the typical values of *BO_%_* computed by the algorithm for different values of hypothesized *d_fil_*. Once the algorithm has enough points to identify the minimum of Ω(*d_fil_*) or, equivalently, the maximum of *BO_%_*, (denoted as *BO_%_max_* in Figure 5) its stopping criteria are satisfied and hence stops, giving in output, for the above-selected *D* and *p*, the optimal value of the strand distance *d_fil_opt_* (Block [S], Figure 4).

The domain of variability of *d_fil_* was hypothesized to range between the following lower and upper bounds: *d_fil_min_* = *D* (which corresponds to have the strands in reciprocal contact) and *d_fil_max_* = 1100 µm which is approximately the average value of the distance of strands utilized by Neufurth et al. [62] and Bartolo et al. [63].

Each finite element analysis had an average duration of 4 hours on an HP XW6600-Intel^®^Xeon^®^DualProcessor E5-5450 3 GHz–32 Gb RAM workstation. Considering that each optimization cycle required about 50 finite element analyses to identify *d_fil_opt_* and considering that five values of *D* (*D* = 400, 500, 600, 700, and 800 µm) and four values of *p* (0.2, 0.5, 1.0, and 1.5 MPa) were hypothesized, one can conclude that the total time to carry out all the analyses conducted in this study is: 4 × 50 × 5 × 4 = 4000 hours. In summary, 5(no. of values of *D*) × 4(no. of values of *p*) = 20 values of *d_fil_opt_* were computed, i.e., 20 scaffold geometries were optimized.

### 2.4. Fabrication of the Optimized Scaffolds

For each of the 20 optimized geometries, three samples of scaffold were fabricated via the FDM technique. With the aim of keeping approximately a cylindrical shape of the deposited filament, an Ultimaker 3 was utilized, adjusting the slice height and the flux of extruded filament into the nozzle. If *D_n_* is the nozzle diameter, *v_n_* is the travel speed of the extrusion head, and *v_h_* is the speed of the filament inside the nozzle, slicing software allows modifying the flow rate of extruded strand according to a flow rate coefficient *f*, usually expressed in terms of percentage

(4) $$D_{n}^{2}v_{n}=D^{2}v_{h}$$

with

(5) $$v_{n}=fv_{h}$$

The deposited filaments are nearly cylindrical if the slice height is equal to *D_n_* , while it is possible to decrease *D* using *f* values lower than 100% and consequently reducing the slice height, according to the Comminal’s model [25], under the hypothesis of negligible effects on the section circular shape. Considering that, commercial extruders are available for Ultimaker 3D printers with a diameter equal to 400 and 800 μm, intermediate values of the strand diameter have been obtained using nozzles 0.4 and 0.8, lowering *f* according to Equations (4) and (5), respectively (Table 2).

As regards the remaining process parameters default values have been exploited: $v_{h}$ = 40 mm/s, extrusion temperature 180° and build plate temperature 50° to lower shrinkage. 

### 2.5. Measurement of the Dimensions of the Fabricated Scaffolds

**Figure 6:**
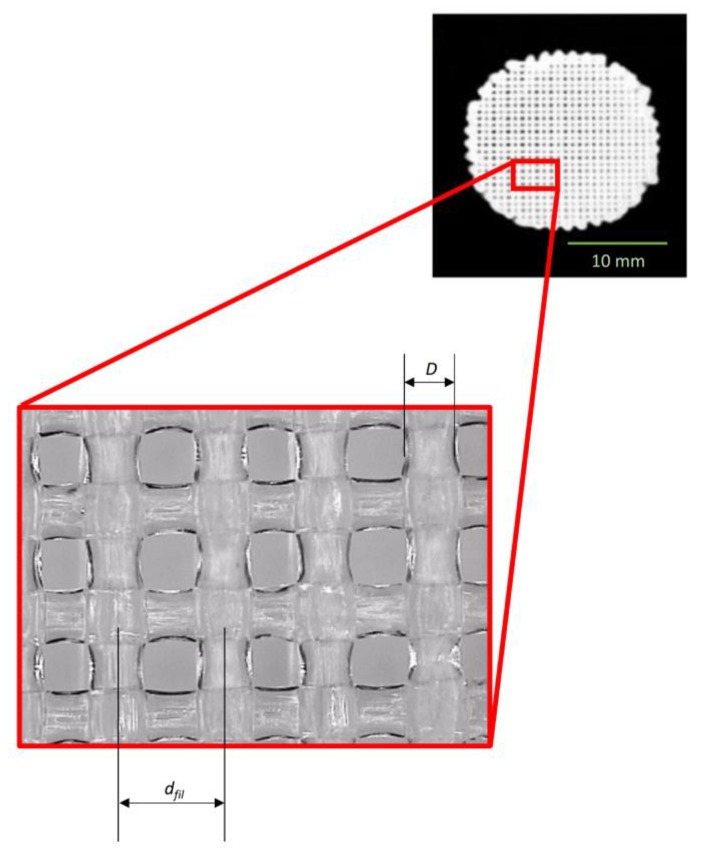
For each scaffold fabricated, the center to center distance between the filaments *d_fil_* and the dimension of the diameter *D* were measured.

A De Meet 400 Coordinate Measuring Machine CMM, by Schut Geometrical Metrology, Germany, was utilized to measure the diameter of strands and their reciprocal distance *d_fil_*. The objective of these measurements was to compare: (i) the distance of filaments actually fabricated and that, denoted as *d_fil_opt_*, optimized with the algorithm above described; (ii) the dimension of the strand diameter actually realized with the nominal one. The CMM is equipped with lenses that can be moved and focused by the user. In detail, *d_fil_* was measured as the center to center distance between the filaments (Figure 6). For each fabricated sample, 10 measurements were taken of *d_fil_* and 10 measurements of *D*.

In order to evaluate the correctness of the hypothesis that an overlap region of 0.1 × *D* exists [57] between two adjacent strands of two consecutive layers, three measurements of this region were carried out for each sample fabricated.

## 3. Results

**Figure 7:**
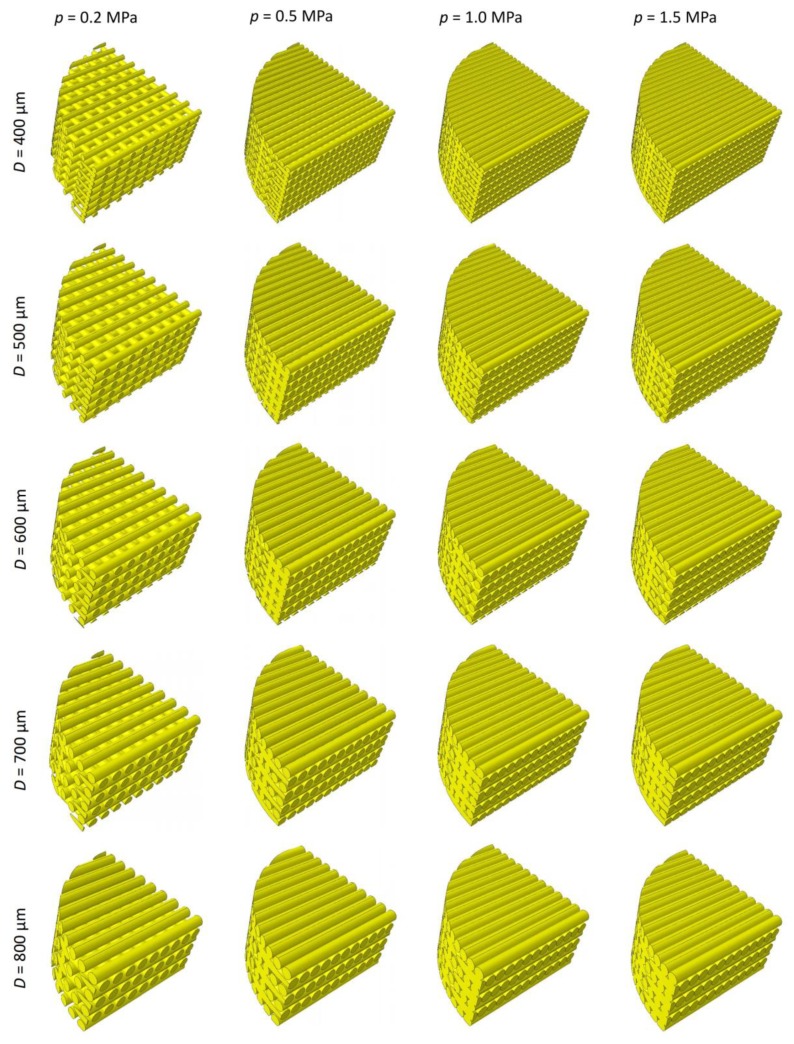
Optimal scaffold geometries predicted by the optimization algorithm for different values of the filament diameter *D* and the load per unit area *p*.

**Figure 8:**
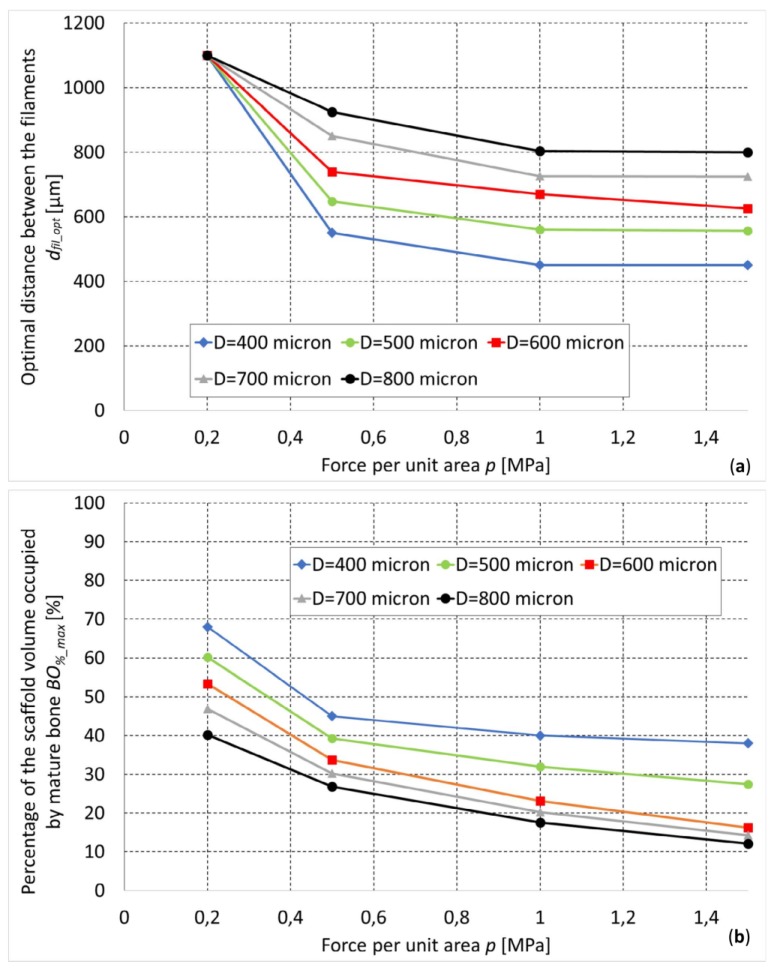
Optimal dimensions of *d_fil_* (**a**) and percentage of the scaffold volume occupied by mature bone (**b**) predicted for different values of the load.

By implementing the optimization algorithm above described, the optimal geometry of the scaffold for different levels of *D* and *p* was computed (Figure 7). As expected, for increasing values of load, decreasing values of the optimal distance *d_fil_opt_* were predicted (Figure 8a). In fact, as the load increases, the biophysical stimulus *S* increases too thus stimulating the formation of ‘softer’ tissues like the cartilage and the fibrous tissue (see Equations (1) and (3)). To prevent this, the algorithm predicts smaller distances between the strands. This leads to an increase in the global stiffness of the scaffold and hence to a decrease in the value of the biophysical stimulus thus stimulating the formation of ‘harder’ tissues such as the bone. Interestingly, for an assigned value of load *p* and for decreasing dimensions of diameter *D*, the algorithm predicts increasing amounts of bone (Figure 8b).

**Figure 9:**
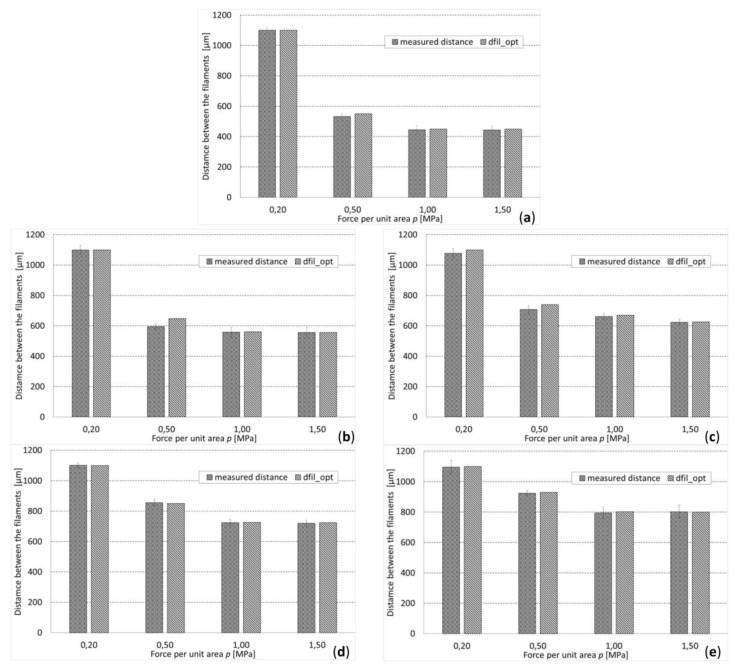
Comparison between measured and optimized distances (between the filaments), for different diameters: (**a**) *D* = 400 µm, (**b**) *D* = 500 µm, (**c**) *D* = 600 µm, (**d**) *D* = 700 µm, (**e**) *D* = 800 µm.

**Figure 10:**
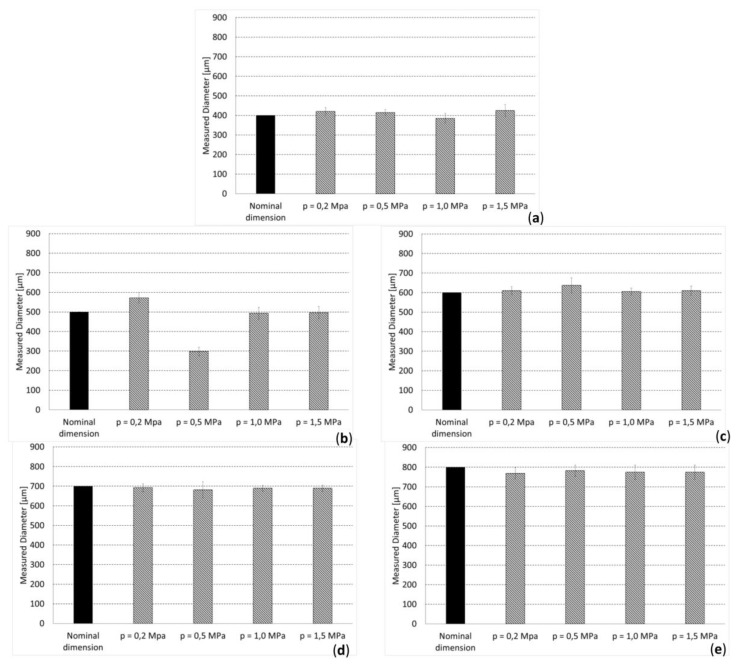
Comparison between measured and nominal dimension of diameter: (**a**) *D* = 400 µm; (**b**) *D* = 500 µm; (**c**) *D* = 600 µm; (**d**) *D* = 700 µm; (**e**) *D* = 800 µm.

The measurements carried out on the overlap region of adjacent filaments revealed an average value of this dimension of (0.12 ± 0.04) × *D*, which demonstrates the reasonable appropriateness of the hypothesis followed [57]. The values of the distance *d_fil_opt_* optimized with the proposed algorithm fell, for almost all the hypothesized values of *p*, within the range [average ± standard_deviation] of the measured dimensions (Figure 9). In general, it appears that the difference between the optimized distances *d_fil_opt_* and the average measured distances never exceeded 8.27% of the optimized distance. Regarding the measured values of the diameter, we noticed that, for *D* = 400 μm and *D* ≥ 600 μm, the measured dimensions are very close to the nominal ones (Figure 10a,c–e)), while for *D* = 500 μm, large differences can be seen (Figure 10b). In fact, for *D* = 400 μm and *D* ≥ 600 μm, the nominal dimension of the diameter fell, almost in all the values of *p* investigated, in the range [average ± standard_deviation] of the measured dimensions. In the case of *D* = 500 μm, instead, the nominal value of the diameter is abundantly out of the above-mentioned range, which indicates that significant fabrication errors are committed.

## 4. Discussion

A feasibility study was conducted aimed to investigate whether the FDM technique can be utilized to fabricate scaffolds designed and optimized to undergo a compression load. A mechanobiology-based optimization algorithm was developed and implemented to determine the optimal distance between the filaments of cylindrical scaffolds for bone tissue engineering. The optimal distance was predicted for different hypothesized values of the load acting on the scaffold and diameter of strands. The scaffolds with the optimized dimensions were hence physically fabricated with the FDM technique and successively measured. The precision guaranteed by the FDM technique was finally evaluated by comparing the measured dimensions with the nominal ones.

This study presents some limitations in the model, the computational mechanobiological algorithm, and the experimental measurements. Regarding the model, first, we hypothesized that the strands of a given layer are aligned and form an angle of 90° with those of the adjacent one. Ideally, the model of the scaffold should include the angle formed between the layers as a design parameter that should be optimized via the mechanobiology-based optimization algorithm above described. 

Second, we hypothesized that an overlap region of 0.1 × *D* exists between two adjacent filaments of consecutive layers [57]. In an ideal model, also this dimension should be included as a design parameter to be optimized by means of fmincon. However, including these two additional design variables would make the computational costs tremendously larger than those spent in this study. Third, it would be interesting to investigate how the proposed optimization algorithm works in the case of more complex loading conditions acting on the scaffold model.

However, the hypothesis of more complex loading conditions would lead to losing the symmetry conditions and hence to the impossibility of using the simplified one-quarter model. Regarding the mechanobiological model, we identified the optimal scaffold geometry based on the values of the biophysical stimulus acting on the granulation tissue, i.e., the tissue that was hypothesized to occupy the volume inside the scaffold pores. In reality, this biophysical stimulus changes in time as the granulation tissue is replaced by the other tissues forming during the regeneration process. In other words, in the mechanobiological model, we did not take into account the variable time. However, the inclusion of the time would increase by at least two orders of magnitude the computational cost required to carry out the analyses. Furthermore, at different compression force p, the optimized value dfil_opt is different. In physiological conditions, the compression force acting on a bone may not be constant but variable. A possible strategy that can be adopted to optimize a scaffold subject to a variable compression load consists in designing functionally graded scaffolds, i.e., scaffolds where the geometric parameters change depending on the specific load value acting in the specific scaffold region. For instance, in the regions where the load acting is higher, a functionally graded scaffold may include strands at a shorter distance, in the regions where, instead, the load is smaller the distance between the filaments may be increased. Such a strategy requires including as many design variables as required to provide an adequate structural response to the variable load and is, therefore, different orders of magnitude more expensive than the approach adopted in this study. Increases in computational power will ultimately allow the investigation of the effect of additional geometric parameters on the optimal scaffold geometry and include different design variables and variable time in the optimization analyses. Regarding the experimental measurements, only two dimensions—the strand diameter and the distance between the filaments—were measured and compared with the corresponding quantities obtained via the mechanobiology-based optimization algorithm. The choice of measuring only these two geometrical parameters is due to the fact that by adjusting the diameter and distance between extruded strands, it is possible to design various topologies with variable values of porosity and therefore to have a wide control of the scaffold micro-architecture [34]. All the other geometric parameters involved in the scaffold designing will certainly play a role less relevant than that played by the distance between the strands and the diameter of the filaments. Furthermore, the proposed optimization algorithm was not validated experimentally. The validation requires a large number of experiments as well as an experimental set-up properly studied and organized to make the experimental conditions equivalent to those hypothesized in the numerical model, which goes beyond the scope of this feasibility study. However, should be clarified that the optimization carried out in this study takes into account only mechanobiological aspects and neglects many other chemical and genetic aspects that certainly affect the differentiation process. Therefore, by ‘optimized design’ we should intend only a design optimized from the mechanobiological point of view. More sophisticated optimizations taking into account the large number of aspects and variables involved in fracture healing should be the object of future studies.

In spite of these limitations, the predictions of the proposed optimization algorithms are consistent with the results of other studies reported in the literature. 

**Figure 11:**
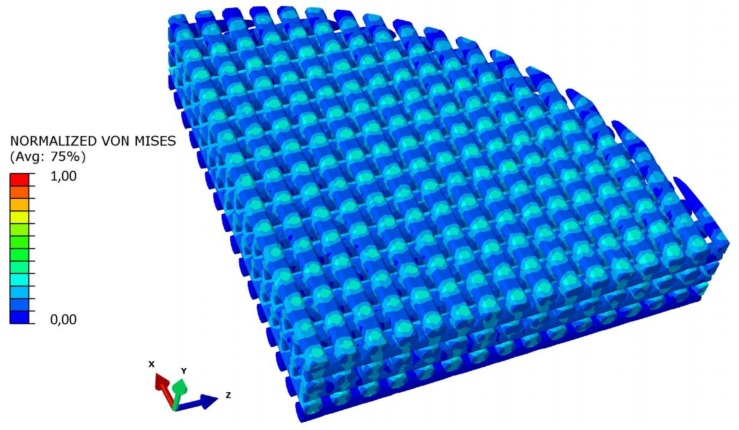
Normalized von Mises stress distribution in a section of the scaffold model. Stress peaks are in alignment with the filaments of the previous layer.

(i) Barba et al. [64] implanted cylindrical scaffolds fabricated with FDM into bone defects generated in the limb of adult beagle dogs. In detail, two scaffold types were implanted, one with filaments of 250 μm and the other with filaments of 450 μm. Interestingly, they found that the amounts of bone formed in scaffolds with filaments of 250 μm are larger than those observed in scaffolds with filaments of 450 μm. This result is consistent with the predictions of the proposed optimization algorithm that found increasing amounts of bone in filaments with decreasing values of the diameter *D* (Figure 8b). 

(ii) The typical distribution of the von Mises stresses within the scaffold model displays the presence of stress peaks in the proximity of the point where the generic strand enters in contact with the strand of the adjacent layer (Figure 11). This same mechanical behavior was reported by Uth et al. [34] who observed peaks of stress in alignment with the filaments of the previous layer. 

(iii) The amounts of bone predicted with the proposed algorithm are comparable with those predicted with other scaffolds based on different unit cell geometries [36,38,40,41]. However, it appears that scaffolds fabricated with the FDM technique allow the formation of amounts of bone significantly smaller than those obtained with other regular, beam-based scaffolds. In general, in scaffolds fabricated with the FDM technique, the amounts of bone predicted to create are approximately 20% lesser than those generated in other scaffolds [41].

The results obtained are consistent with the physics of the problem. As the load increases, the biophysical stimulus *S* increases too and with it, the percentage of the scaffold volume occupied by ‘soft’ tissues such as the cartilage and the fibrous tissue. To counterbalance this tendency, the optimization algorithm tends to decrease the distance between the filaments hence making the scaffold stiffer. The increase in stiffness leads to decreasing levels of *S* and hence to the formation of harder tissues such as the immature and the mature bone. For very high values of load, the optimal distance *d_fil_opt_* tends, asymptotically, towards the dimension of the filament diameter *D* (Figure 8a). The optimal distance between the filaments *d_fil_opt_* was determined for different values of load and for different assigned values of diameter *D* (Figure 8b). In general, it appears that using smaller diameters, for a fixed value of load *p*, leads to the formation of larger amounts of bone (Figure 8b). Therefore, if one can choose the nozzle diameter (i.e., if the FDM machine is equipped with nozzles of different diameter), to which the strand diameter is strictly related, should prefer the smaller diameters.

The dimensions measured on the samples were compared with the nominal one, in order to assess the accuracy guaranteed by FDM in the fabrication of scaffolds for bone tissue engineering. For almost all the hypothesized values of load per unit area p, the optimal dimensions dfil_opt fell within the range (average ± standard_deviation) of the measured distances. This leads us to conclude that the FDM is suited to reproduce with high accuracy the designed and optimized distance between the filaments dfil_opt. Furthermore, the values of the standard deviation of the measured distances (between the filaments) never exceeded 6.02% of the optimized dimension, which indicates a reasonably small dispersal of data and hence a rather high reproducibility of the fabrication process, in terms of the distance between the filaments. Regarding the measured dimensions of the filament diameter, we found that the diameters are well reproduced in the case of D = 400 μm and D ≥ 600 μm, but large reproduction errors are made when D = 500 μm. This behavior can be justified with the argument that the strategy of using different flow percentages of PLA to have filaments with different diameters presents a lower limit below which the quality of the filament diameter decreases significantly. Indeed, 500 μm is the diameter that mostly differs with respect to that of the nozzles utilized to extrude the filaments. We can hypothesize that when the material flow is significantly smaller than 100%, the nozzle does not fill correctly and hence the filament cannot form properly. This leads to the deposition of filaments with dimensions significantly different with respect to the nominal ones. However, this limitation can be easily overcome by equipping the FDM machine with a greater number of nozzles. In general, in ideal conditions, to minimize the fabrication errors, nozzles with the same diameter of the strands to be fabricated should be utilized.

## 5. Conclusions

We conducted a feasibility study which aimed to investigate the potentialities of the FDM technique to be used for the fabrication of scaffolds designed and optimized with mechanobiological algorithms. The present article is the first study ever reported in the literature where the geometry of scaffolds fabricated with the FDM technique is optimized via a mechanobiology-based optimization algorithm. In detail, the optimal distance between the filaments was predicted in function of the filament diameter and of the load acting on the scaffold. The designed and optimized scaffolds have been fabricated and measurements on the dimensions of the samples realized were carried out. We found that the difference between the average dimensions of the fabricated scaffolds and the nominal ones never exceeded 8.27% of the nominal dimension, which demonstrates the rather good accuracy of the FDM technique in reproducing the distance between the filaments. Furthermore, the values of the standard deviation of the acquired distances (between the filaments) never exceeded 6.02% of the optimized dimension, which indicates a reasonably small dispersal of data and hence a rather high reproducibility of the fabrication process in terms of distance between the filaments. However, we found that large reproduction errors are made on the filament diameter when the filament diameter to be realized differs significantly with respect to the nozzle diameter. 

In conclusion, we can state that the FDM technique is suitable to build accurate scaffold samples only in the cases where the filament diameter is close to the nozzle diameter. Conversely, when a large difference exists, large fabrication errors can be made on the diameter of the filaments. In general, the scaffolds realized with the FDM technique were predicted to stimulate the formation of amounts of bone smaller than those that can be obtained with other regular beam-based scaffolds. 

## Figures and Tables

**Table 1:** Material properties utilized in the model of scaffold and granulation tissue

Material Properties	Scaffold	Granulation Tissue
Young’s modulus (MPa)	2300	0.2
Poisson’s ratio	0.3	0.167
Permeability (m^4^/N/s)	1 × 10^−14^	1 × 10^−14^
Porosity	0.5	0.8
Bulk modulus grain (MPa)	13920	2300
Bulk modulus fluid (MPa)	2300	2300

**Table 2:** Values of the flow rate *f* utilized to obtain different strand diameters

Fabricated Strand Diameter *D* (μm)	Nozzle Diameter *D_n_* (μm)	Flow Rate *f* (%)
400	400	100
500	800	39
600	800	56
700	800	76
800	800	100
